# Function and Immunogenicity of Gene-corrected iPSC-derived Hepatocyte-Like Cells in Restoring Low Density Lipoprotein Uptake in Homozygous Familial Hypercholesterolemia

**DOI:** 10.1038/s41598-019-41056-w

**Published:** 2019-03-18

**Authors:** Hirofumi Okada, Chiaki Nakanishi, Shohei Yoshida, Masaya Shimojima, Junichiro Yokawa, Masayuki Mori, Hayato Tada, Tsuyoshi Yoshimuta, Kenshi Hayashi, Tomoyoshi Yamano, Rikinari Hanayama, Masakazu Yamagishi, Masa-aki Kawashiri

**Affiliations:** 1 Department of Cardiovascular and Internal Medicine, Kanazawa University Graduate School of Medicine, Takara-machi 13-1, Kanazawa, Ishikawa 920-8641 Japan; 2 Department of Immunology, Kanazawa University Graduate School of Medicine, Takara-machi 13-1, Kanazawa, Ishikawa 920-8640 Japan; 3 Department of Human Sciences, Osaka University of Human Sciences, 1-12-13 Shoya, Settsu, Osaka 566-8501 Japan

## Abstract

Gene correction of induced pluripotent stem cells (iPSCs) has therapeutic potential for treating homozygous familial hypercholesterolemia (HoFH) associated with low-density lipoprotein (LDL) receptor (LDLR) dysfunction. However, few data exist regarding the functional recovery and immunogenicity of *LDLR* gene-corrected iPSC-derived hepatocyte-like cells (HLCs) obtained from an HoFH patient. Therefore, we generated iPSC-derived HLCs from an HoFH patient harbouring a point mutation (NM_000527.4:c.901 G > T) in exon 6 of *LDLR*, and examined their function and immunogenicity. From the patient’s iPSCs, one homozygous gene-corrected HoFH-iPSC clone and two heterozygous clones were generated using the CRISPR/Cas9 method. Both types of iPSC-derived HLCs showed recovery of the function of LDL uptake in immunofluorescence staining analysis. Furthermore, these gene-corrected iPSC-derived HLCs showed little immunogenicity against the patient’s peripheral blood mononuclear cells in a cell-mediated cytotoxicity assay. These results demonstrate that LDL uptake of iPSC-derived HLCs from HoFH can be restored by gene correction without the appearance of further immunogenicity, suggesting that gene-corrected iPSC-derived HLCs are applicable to the treatment of HoFH.

## Introduction

Familial hypercholesterolemia (FH), a common and severe monogenic hyperlipidaemia that results in early death due to premature coronary and systemic atherosclerosis^1–3^, is associated with mutations or defects in the gene encoding the low-density lipoprotein (LDL) receptor (*LDLR*)^1,2,4^. Cholesterol-lowering agents including statins and PCSK9 inhibitors have been developed^5^, but these drugs are minimally effective for homozygous FH (HoFH), where a functional LDLR is completely lacking, although invasive procedures such as LDL apheresis and liver transplant are effective for reducing LDL levels in these patients^6–11^. In terms of genetic approaches, the effect of virus-mediated LDLR gene transfer to reduce LDL levels is not persistent^7,8^. Therefore, an alternative therapy for restoring normal LDLR function is required.

Induced pluripotent stem cells (iPSCs) can differentiate into all types of cells including hepatocyte-like cells (HLCs)^12–14^ and cardiomyocytes^15,16^. Indeed, iPSC-derived HLCs can be used as a model of FH^17–19^. Under these conditions, the method of clustered regularly interspaced short palindromic repeats/CRISPR-associated nuclease 9 (CRISPR/Cas9) contributes to enhancing the effective gene correction for a long time^20–24^. However, few studies have evaluated the function and immunogenicity of iPSCs-derived HLCs against peripheral blood mononuclear cells (PBMCs), although the gene correction of iPSCs from fibroblasts obtained from a patient with HoFH has been reported^25^. Here, we generated iPSC-derived HLCs from lymphocytes of an HoFH patient and examined the restoration of LDL uptake after gene correction. We also examined the immunogenicity of iPSC-derived HLCs, with or without gene correction, against the patient’s PBMCs.

## Results

### Generation and Gene Correction of iPSCs

**Figure 1:**
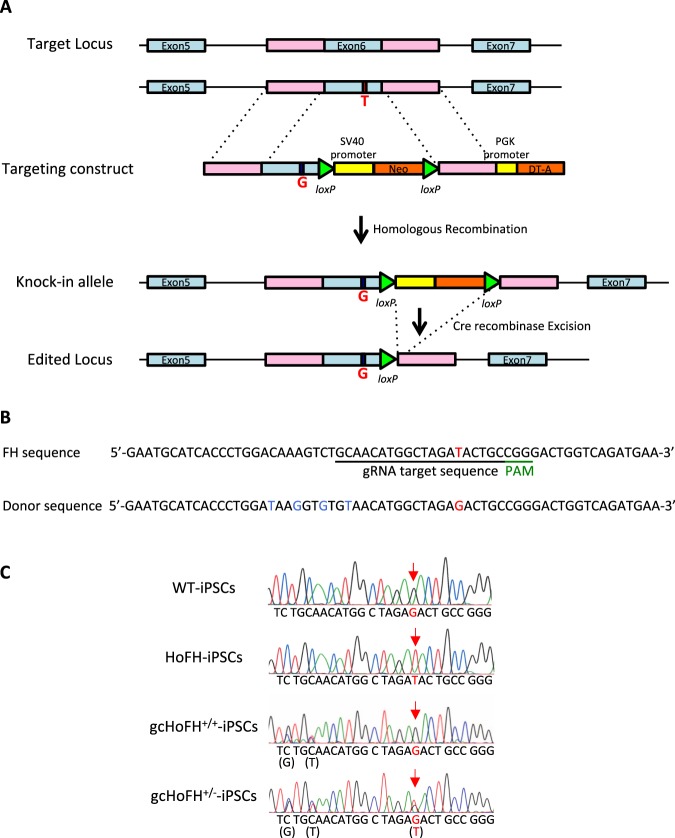
Gene correction of iPSCs using the CRISPR/Cas9 method. (**A**) Schematic overview of the gene-targeting strategy for the human *LDLR* mutation. After introduction of a double-strand DNA break at *LDLR* exon 6 near the point mutation, a normal amino acid sequence along with a neomycin-selection cassette flanked by two loxP sites was inserted using a homologous recombination vector. Finally, after selection and sequencing of the targeted vector, the selection cassette was excised by transient Cre expression. (**B**) An sgRNA with a 23-base pair target sequence corresponding to bases adjacent to the mutation site in exon 6 of human *LDLR* was designed. A donor sequence, containing a T > G correction for the point mutation, was used as a template for the homology-directed repair process induced by Cas9 cleavage. Blue characters indicate silent mutations. SV40, simian virus; Neo, neomycin resistance gene; PGK phosphoglycerine kinase; DT-A diphtheria toxin A; PAM, protospacer adjacent motif. (**C**) Genomic sequencing showing retention of the *LDLR* mutation in the HoFH-iPSC line and correction of the target sequence in the gcHoFH-iPSC lines (arrows). Wild-type-derived iPSCs (WT-iPSCs), homozygous FH-derived iPSCs (HoFH-iPSCs), homozygous gene-corrected HoFH-iPSCs (gcHoFH^+/+^-iPSCs), and heterozygous gene-corrected HoFH-iPSCs (gcHoFH^+/−^-iPSCs).

A schematic overview of the gene-targeting strategy for the human LDLR mutation is shown in Fig. 1. Using immunostaining and PCR, we confirmed that both wild-type-derived iPSCs (WT-iPSCs) obtained from a healthy volunteer and HoFH-derived iPSCs (HoFH-iPSCs) expressed pluripotency markers after reprograming of the T cells to iPSCs (Supplementary Fig. 1A,B). In addition, these cells spontaneously differentiated into three germ layers (Supplementary Fig. 1C). A short tandem repeat (STR) analysis was performed to show that the STR in the D12S391 locus of HoFH-iPSCs was matched with donor PBMCs (Supplementary Fig. 2).

Next, we isolated 16 clones using the neomycin selection and limiting dilution method after transfection with CRISPR sgRNA, Cas9 nuclease, and donor plasmid. Under these conditions, PCR revealed that 13 clones had the knock-in allele (Supplementary Fig. 3). After Cre/loxP-mediated excision of the neomycin resistance expression unit, we obtained one homozygous gene-corrected HoFH-iPSC (gcHoFH^+/+^-iPSC) clone and two heterozygous gene-corrected HoFH-iPSC (gcHoFH^+/−^-iPSC) clones. We again confirmed both the presence of pluripotency markers in these cells and differentiation of the three germ layers (Supplementary Fig. 1A–C). Genomic sequencing showed retention of the *LDLR* mutation in HoFH-iPSCs and correction of the target sequence in gcHoFH-iPSCs (Fig. 1C, arrows).

### Generation of HLCs from iPSCs

Morphologically, the iPSCs gradually assumed a cobblestone or polygonal shape with a lower nucleus to cytoplasm ratio during differentiation. In the hepatic endoderm, the cells showed canaliculi-like structures with a dark cytoplasm. Lipid vesicles and multi-nucleated cells were observed after 25 days of differentiation (Supplementary Fig. 4A). Immunostaining for hepatic markers such as albumin and α-1-antitrypsin confirmed differentiation of iPSCs to HLCs (Supplementary Fig. 4B). RT-PCR of differentiation markers showed the expression of hepatocyte nuclear factor 4-α, α-1-fetoprotein, and albumin, indicating the occurrence of transition in these cells (Supplementary Fig. 4C).

### LDLR Expression and LDL Uptake in iPSC-derived HLCs

**Figure 2:**
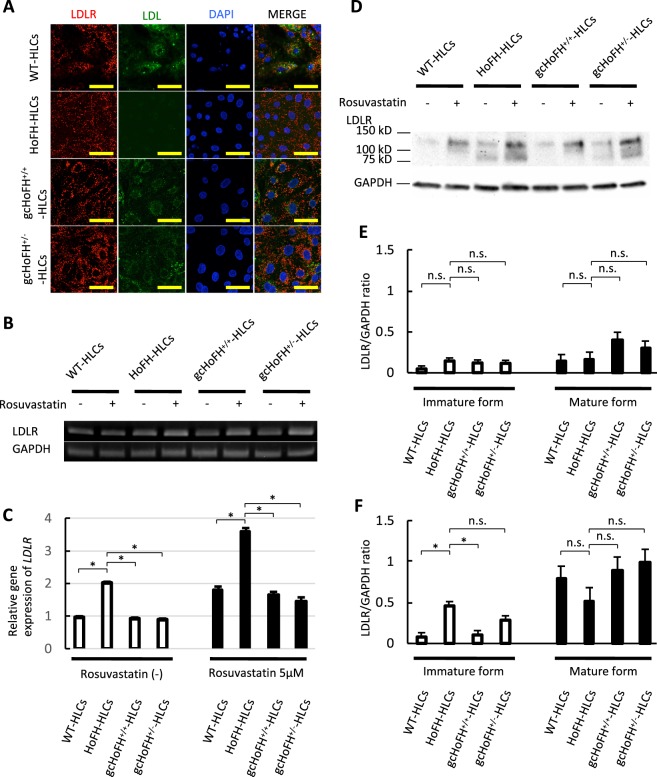
LDLR expression and LDL uptake in iPSC-derived HLCs. (**A**) Immunofluorescence staining showing the presence of LDLR in the membrane and cytoplasm of WT-iPSC-derived hepatocyte-like cells (WT-HLCs), HoFH-iPSC-derived hepatocyte-like cells (HoFH-HLCs), gcHoFH^+/+^-iPSC-derived hepatocyte-like cells (gcHoFH^+/+^-HLCs), and gcHoFH^+/−^-iPSC-derived hepatocyte-like cells (gcHoFH^+/−^-HLCs). There was no apparent receptor-mediated internalization of BODIPY-labelled LDL in HoFH-HLCs, although this function was preserved in WT-HLCs, gcHoFH^+/+^-HLCs, and gcHoFH^+/−^-HLCs (scale = 50 µm). (**B**) RT-PCR assay, and (**C**) real-time PCR analysis for *LDLR* levels without (white bar) or with (black bar) rosuvastatin treatment. Statistical significance was defined as *p < 0.05. (**D**) Before treatment with rosuvastatin, mature LDLR was expressed in WT-HLCs and gcHoFH^+/+^-HLCs. HoFH-HLCs and gcHoFH^+/−^-HLCs expressed both the immature and the mature form of LDLR. Rosuvastatin treatment enhanced LDLR expression in all cell lines. (**E**,**F**) Quantitative evaluation of LDLR protein by western blotting without (**E**) and with (**F**) rosuvastatin treatment. Mature and immature forms of LDLR were not significantly different in all cell lines (**E**). On the other hand, the amount of the immature form was significantly larger in HoFH-HLCs and gcHoFH^+/−^-HLCs than in WT-HLCs and gcHoFH^+/+^-HLCs (**F**). Statistical significance was defined as *p < 0.05. Bars show mean ± SE. n.s. = not significant.

Immunofluorescence staining in iPSC-derived HLCs showed the presence of LDLR in the membrane and cytoplasm of WT-iPSC-derived HLCs (WT-HLCs), HoFH-iPSC-derived HLCs (HoFH-HLCs), gcHoFH^+/+^-iPSC-derived HLCs (gcHoFH^+/+^-HLCs), and gcHoFH^+/−^-iPSC-derived HLCs (gcHoFH^+/−^-HLCs) (Fig. 2A, Supplementary Fig. 5). Under these conditions, there was no apparent receptor-mediated internalization of BODIPY-labelled LDL in HoFH-HLCs, although this function was preserved in WT-HLCs. Importantly, gcHoFH^+/+^-HLCs and gcHoFH^+/−^-HLCs also showed LDL uptake ability (Fig. 2A). By double immunostaining with ER-GFP and LDLR, LDLR was observed both on the cell surface and in the cytoplasm in all lines of HLCs, and colocalization was observed in HoFH-HLCs (Supplementary Fig. 6). Real-time PCR analysis demonstrated that *LDLR* mRNA levels were downregulated in gcHoFH^+/+^-HLCs and gcHoFH^+/−^-HLCs as compared with HoFH-HLCs with or without statin treatment (Fig. 2B,C).

Western blotting detected the mature form of LDLR (130 kDa) in all lines of HLCs, particularly in the presence of 5 µM rosuvastatin (Wako Chemicals, Osaka, Japan) (Fig. 2D). By contrast, the immature form of LDLR (85 kDa) was detected in HoFH-HLCs and gcHoFH^+/−^-HLCs. Quantitative evaluation of LDLR protein by western blotting showed that the mature and immature forms of LDLR were not significantly different in all cell lines (Fig. 2E). On the other hand, the immature form was present in significantly larger amounts in HoFH-HLCs than in WT-HLCs and gcHoFH^+/+^-HLCs (Fig. 2F).

### Restored Functions of LDLR in gcHoFH-HLCs

**Figure 3:**
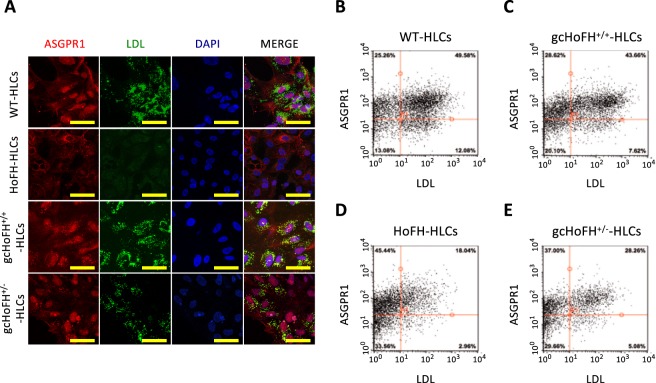
Immunostaining and flow cytometric analysis for determining restored functions of LDLR. (**A**) Immunostaining for ASGPR1 and LDL (scale = 50 µm). WT-iPSC-derived hepatocyte-like cells (WT-HLCs), gcHoFH^+/+^-iPSC-derived hepatocyte-like cells (gcHoFH^+/+^-HLCs), and gcHoFH^+/−^-iPSC-derived hepatocyte-like cells (gcHoFH^+/+^-HLCs) showed a high uptake of fluorescence-labelled LDL, whereas this uptake was impaired in HoFH-iPSC-derived hepatocyte-like cells (HoFH-HLCs). (**B**–**E**) Flow cytometry showing LDL uptake in WT-HLCs (**B**, 49.6%), gcHoFH^+/+^-HLCs (**C**, 43.7%), HoFH-HLCs (**D**, 18.0%), and gcHoFH^+/−^-HLCs (**E**, 28.3%).

We confirmed the expression of anti-asialoglycoprotein receptor 1 (ASGPR1) (hepatic surface marker) in all iPSC-derived HLCs and the impairment in LDL uptake in HoFH-HLCs using immunofluorescence staining (Fig. 3A, Supplementary Fig. 7) and flow cytometry (Fig. 3B–E). After normalization to the number of ASGPR1-positive cells, LDL uptake in gcHoFH^+/+^-HLCs (43.7%) was found to be higher than that in HoFH-HLCs (18.0%), and was similar to that in WT-HLCs (49.6%) (Fig. 3B–D). Under these conditions, gcHoFH^+/−^-HLCs demonstrated intermediate uptake of LDL (28.3%) (Fig. 3E).

### Immunogenicity of iPSC-derived HLCs

**Figure 4:**
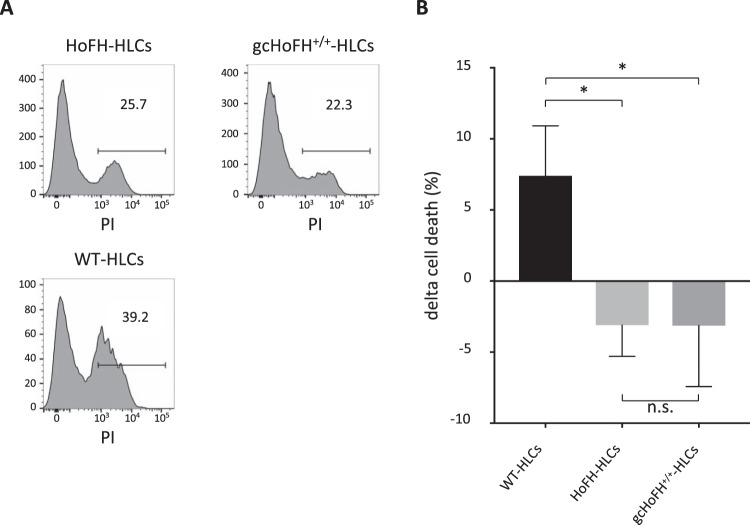
Analysis of autoimmune reaction by cell-mediated cytotoxicity assay. (**A**) Percentage of dead cells determined by flow cytometry. (**B**) Changes in percent cell death in each iPSC-derived HLC cell line. Statistical significance was defined as *p < 0.05. n.s. = not significant.

Next, we examined cellular immunogenicity by using a cell-mediated cytotoxicity assay in which immune response-induced target cell death was evaluated^26^. Percent cell death in HoFH-HLCs and gcHoFH^+/+^-HLCs (Fig. 4A, upper) was lower than that in WT-HLCs (Fig. 4A, lower). Delta cell death was 7.2 ± 3.2% in WT-HLCs. Interestingly, it was −3.1 ± 2.2% and −3.2 ± 3.6% in HoFH-HLCs and gcHoFH^+/+^-HLCs, respectively (p < 0.05, Fig. 4B). This suggests that immunogenicity might be minimized in gcHoFH^+/+^-HLCs and HoFH-HLCs as compared with WT-HLCs.

## Discussion

Here, we demonstrated that iPSCs could be generated from lymphocytes of a patient with HoFH and differentiated into HLCs with characteristics of the lipid metabolic disorder of the patient. Gene correction using the CRISPR/Cas9 method restored LDL uptake ability in HoFH-HLCs to levels similar to those of WT-HLCs, and HoFH-HLCs and gcHoFH^+/+^-HLCs showed little immunogenicity against the patient’s PBMCs *in vitro*.

The most common cause of the HoFH phenotype is a mutation in both *LDLR* alleles^3^. The patient studied here harboured a point mutation in exon 6 of both *LDLR* alleles. Although exon 6 encodes the ligand-binding domain of LDLR^26^, there are no reports on the mutation classes of the c.901 G > T point mutation. We successfully generated iPSCs from this patient and obtained iPSC-derived HLCs. Consequently, the iPSC-derived HLCs expressed an LDLR with impaired LDL uptake. Previously, we reported that the LDLR activity of fibroblasts derived from this patient was approximately one-tenth of that of fibroblasts from a healthy subject^27^. These findings are consistent with the LDL uptake of the iPSC-derived HLCs presented here. LDLR protein is usually glycosylated in the endoplasmic reticulum and transported to the Golgi, where it is converted into mature LDLR, and immature LDLR proteins are thought to be insufficiently modified. In present study, western blotting showed that HoFH-HLCs expressed both mature and immature LDLR proteins, which suggest that c.901 G > T is a class IIB mutation^28^.

Using the single CRISPR/Cas9 transfection method^29^, we generated not only gcHoFH^+/−^-iPSCs but also gcHoFH^+/+^-iPSCs that expressed mature LDLR with LDL uptake function, although the gcHoFH^+/+^-iPSC clone was determined to be heterozygous for all intentionally inserted silent mutations. A possible explanation is that the homologous recombination-mediated knock-in occurred only in a small range of donor sites, as observed in another study^25^. One-base substitution to the wild-type sequence with nonhomologous end joining might occur in one allele, although the precise mechanism of this phenomenon is still unclear.

One of the advantages of the present study is that the iPSC-derived HLCs were obtained not from patient fibroblasts but from peripheral lymphocytes, which might be less invasive than previous procedures to analyse their function and immunogenicity. Under our conditions, the capacity of LDL uptake after homozygous gene correction seemed to be similar to that of WT-HLCs. In addition, it is interesting that the patient’s iPSC-derived HLCs after gene correction exhibited little immunogenicity, similar to that observed before gene correction, whereas WT-HLCs from the healthy volunteer showed significant immunogenicity against the patient’s PBMCs. It is intriguing to point out that an auto-immune response was similarly not observed after hepatic cell transplantation with gene therapy^30^. This is the first study to perform a cell-mediated cytotoxicity assay for investigating the utility of gene-corrected iPSCs as autologous transplanted cells *in vitro*. Changes in the LDLR steric structure caused by gene correction may not have affected overall immunogenicity in either a previous study^30^ or the present study. This suggests that the gcHoFH-HLCs described here are suitable for transplantation without aggressive immune suppressive therapy.

The present study has several limitations. First, we did not quantify the LDL uptake ability of the gcHoFH-HLCs. Therefore, the extent to which iPSC-derived HLCs can assimilate LDL as compared with human hepatocytes remains unclear. However, the fact that gcHoFH^+/+^-HLCs showed LDL uptake equal to that in WT-HLCs suggests the functional restoration of these cells. Second, we did not further evaluate the immune response against the newly-developed LDLR in gcHoFH-HLCs, although we confirmed little immune response by human leukocyte antigen mismatch. However, as observed in our study, total cell damage might be minimized when the patient’s iPSC-derived HLCs are used. Finally, we did not investigate whether the gene correction affected insertional oncogenesis. A previous study demonstrated few possibilities of insertional mutagenesis in oncogenes using CRISPR/Cas9 gene correction^31^. However, this might vary with the type of transplanted cell, insertion location, and method. Further *in vivo* studies are necessary to address these issues.

## Conclusion

LDL uptake ability of iPSC-derived HLCs can be restored by CRISPR/Cas9-mediated gene correction without altering immunogenicity. We suggest that gene correction using the CRISPR/Cas9 system can be used to treat HoFH patients with LDLR dysfunction.

## Material and Methods

### Generation of iPSCs from the HoFH Patient’s T Cells

This study was reviewed and approved by the Research Ethics Committee of Kanazawa University (Kanazawa, Japan), and written informed consent was obtained from the patient and the volunteer. All procedures were conducted in accordance with the Helsinki Declaration of 1996. The animal study was approved by the Animal Care and Use Committee of Kanazawa University. Use of animals was conducted according to the “Basic Guidelines for Conduct of Animal Experiments” published by the Ministry of Health, Labor, and Welfare, Japan.

T cells were harvested from a 66-year-old male patient with HoFH and a healthy volunteer. The patient’s profile has been reported elsewhere^27^. This patient has a point mutation (NM_000527.4:c.901 G > T) in exon 6 of *LDLR*. From these T cells, iPSCs were generated using a published protocol^32^. The pluripotent potential of iPSCs was assessed by their ability to spontaneously differentiate into three germ layers (endoderm, mesoderm, and ectoderm) *in vitro* as described previously^33^. The generated iPSCs were maintained on mitomycin C-inactivated mouse embryonic fibroblasts (MEFs) in Dulbecco’s modified Eagle’s medium/nutrient mixture F-12 Ham (DMEM/F12, Sigma-Aldrich, MO, USA) supplemented with 1 mM L-glutamine (Invitrogen, CA, USA), 1 mM non-essential amino acids (NEAAs) (Sigma-Aldrich), 1% penicillin/streptomycin (pen/strep), 20% knockout serum replacement (Invitrogen), 0.1 mM β-mercaptoethanol (Sigma-Aldrich), and 4 ng/ml of basic fibroblast growth factor (bFGF; Wako Chemicals). The iPSC culture medium was changed every 2 days, and the cells were passaged using 1 mg/ml of collagenase IV (Invitrogen) every 7 days.

### Differentiation of iPSCs into Hepatocyte-like Cells

Differentiation of iPSCs toward the hepatic lineage was performed according to the method described by Hannan *et al*.^34^. Briefly, iPSCs were split (day 0) and maintained for 48 h in chemically defined media (CDM)-polyvinyl alcohol (PVA) medium consisting of 500 mg of PVA (Sigma-Aldrich) dissolved in 250 ml of DMEM/F12, GlutaMAX (Invitrogen), 250 ml of Iscove’s modified Dulbecco’s medium (IMDM) (Invitrogen), 5 ml of concentrated lipids (Invitrogen), 20 µl of thioglycerol (Sigma-Aldrich), 350 µl of insulin (10 mg/ml; Roche, Basel, Switzerland), 250 µl of transferrin (30 mg/ml; Roche), 1% pen/strep supplemented with 10 ng/ml of activin A (Shenandoah, PA, USA), and 12 ng/ml of bFGF. On day 2, the cells were differentiated in CDM-PVA medium supplemented with 100 ng/ml of activin A, 80 ng/ml of bFGF, 10 ng/ml of bone morphogenetic protein 4 (BMP4; R&D Systems, MN, USA), 10 µM LY-294002 (Promega, WI, USA), and 3 µM Stemolecule CHIR99021 (StemGent, MA, USA). On day 3, the cells were differentiated in CDM-PVA medium supplemented with 100 ng/mL of activin A, 80 ng/mL of bFGF, 10 ng/ml of BMP4, and 10 µM LY-294002.

On day 4, the cells were differentiated in Roswell Park Memorial Institute (RPMI)-1640 medium containing GlutaMAX, 2% B-27 supplement containing insulin (Invitrogen), 1% NEAAs, 1% pen/strep, 100 ng/ml of activin A, and 80 ng/ml of bFGF. On days 5–7, the cells were differentiated in RPMI medium supplemented with 50 ng/ml of activin A. On days 8–12, the cells were differentiated in RPMI-B27 medium containing 20 ng/ml of BMP4 and 10 ng/ml of FGF10 (Autogen Bioclear, Nottingham, UK). After day 12, the cells were differentiated in hepatocyte basal medium (HBM; Lonza, Basel, Switzerland) supplemented with 30 ng/ml oncostatin M (R&D Systems) and 50 ng/ml of hepatocyte growth factor (PeproTech, NJ, USA) with a medium change every other day. iPSC-derived HLCs were treated for 24 h with 5 µM rosuvastatin or vehicle (0.025% dimethyl sulfoxide) before RNA isolation or protein extraction.

### Genomic Sequence of iPSCs

Genomic DNA was isolated from iPSCs using a Gentra Puregene Cell kit (Qiagen, Venlo, Netherlands), and the region encoding exon 6 of LDLR was amplified by PCR. The PCR products were electrophoresed on a 2% agarose gel and purified using a Wizard SV gel and PCR clean-up system (Promega). The purified PCR product was sequenced using a BigDye® Terminator (v1.1 or v3.1) (Applied Biosystems, CA, USA) and an Applied Biosystems 3130 Genetic Analyzer (Applied Biosystems).

### Short tandem repeats-PCR analysis

Short tandem repeats (STRs) are tandemly repeated simple sequences of a few bases in length that vary in number of repeat units among alleles from different individuals^35^. STR-PCR analysis was performed to facilitate individual identification. Briefly, PCR amplification was performed for the STR in the D12S391 locus using AmpliTaq Gold (Thermo Fisher Scientific). The primer sequences were forward, AACAGGATCAATGGATGCAT, and reverse, TGGCTTTTAGACCTGGACTG. The forward primers were labelled at the 5′ end with 6-carboxyfluorescein. Amplification was performed in a thermal cycler (ASTEC, Fukuoka, Japan) for 30 cycles. The PCR products (1 μl) were mixed with 0.3 μl of size standards (GeneScan 500LIZ dye Size Standard; Thermo Fisher Scientific), 23.5 μl of Hi-Di formamide (Thermo Fisher Scientific), and 1.2 of μl nuclease-free water. DNA bands were detected using an ABI PRISM 310, and peak patterns were visualized using GeneMapper software (Applied Biosystems).

### Gene expression analysis

Total RNA was isolated from cells using an RNAeasy kit (Qiagen). For real-time PCR analysis, cDNA was synthesized using SuperScript III reverse transcriptase and oligo (dT) primers (Invitrogen). Primers used for amplification are listed in Supplemental Table 1. Real-time PCR was conducted using the Power SYBR® Green PCR Master Mix (Applied Biosystems). Real-time PCR for LDLR and glyceraldehyde-3-phosphate dehydrogenase (GAPDH) was performed using an Mx3000P QPCR system (Agilent Technologies, CA, USA), and the cycling conditions were 10 min at 95 °C, followed by 45 cycles of 30 s at 95 °C and 1 min at 60 °C. Cycle threshold was calculated under default settings using real-time sequence detection software (Agilent Technologies). The expression levels of *LDLR* were normalized to those of *GAPDH*.

### Immunostaining

Cells were fixed in 4% formaldehyde for 15 min, permeabilized with phosphate-buffered saline (PBS) containing 0.1% Triton X-100 for 15 min, and blocked with 1% bovine serum albumin (BSA; Sigma-Aldrich) in PBS for 30 min. Next, the cells were incubated for 1 h at room temperature with the following primary antibodies diluted in blocking solution: Nanog (1:100; ReproCELL, Kanagawa, Japan), OCT3/4 (1:100; Santa Cruz Biotechnology, CA, USA), SSEA-4 (1:100; Santa Cruz Biotechnology), TRA-1-60 (1:100; Santa Cruz Biotechnology), TRA-1-81 (1:100; Santa Cruz Biotechnology), nestin (1:100; Sigma-Aldrich), SOX17 (1:100; R&D Systems), smooth muscle actin (SMA; 1:100; Dako, Glostrup, Denmark), α-1-antitrypsin (A1AT; 1:100; Dako), albumin (ALB; 1:100; R&D Systems), LDLR (1:100; Abcam, Cambridge, UK), rabbit IgG polyclonal isotype control (Thermo Fisher Scientific) for A1AT, mouse IgG2a isotype control (R&D Systems) for ALB, rabbit IgG monoclonal isotype control (abcam) for LDLR, and mouse IgG2b isotype control (Thermo Fisher Scientific) for ASGPR1. In the ER localization experiment only, cells were treated for 24 h with a green fluorescent protein fused to an ER retention signal (CellLight ER-GFP BacMam 2.0, Invitrogen) before immunostaining according to the manufacturer’s instructions.

Cells were washed three times with PBS for 5 min each and then incubated for 1 h at room temperature with appropriate secondary antibodies (Alexa Fluor 488 series (1:1000; Invitrogen) and Alexa Fluor 568 series (1:1000; Invitrogen)) diluted in blocking solution. Nuclei were stained using 4′,6-diamidino-2-phenylindole (DAPI) (1:1000; Dojindo Laboratories, Kumamoto, Japan) for 5 min. Cells were washed three times with PBS for 5 min each and then imaged using a fluorescence microscope (BZ-9000, Keyence, Osaka, Japan).

### Fluorescence-labelled LDL uptake assay

The immunofluorescence LDL uptake assay was performed according to the modified protocol of Cayo *et al*.^18^. iPSC-derived HLCs were washed three times with ice-cold PBS, and then incubated in ice-cold HBM containing 5 µg/ml of BODIPY^®^ FL LDL (Invitrogen) at 37 °C for 4 to 6 h. After termination of the assay, iPSC-derived HLCs were washed with PBS and treated as described below for flow cytometry. Fixation and permeabilization of iPSC-derived HLCs for imaging were performed as described above. Cells were incubated for 1 h at room temperature with ASGPR1 antibody diluted in 1% BSA in PBS (1:100; Santa Cruz Biotechnology). The cells were washed twice with PBS for 5 min each, and then incubated for 1 h at room temperature with secondary antibody (Alexa Fluor 568; 1:1000; Invitrogen) diluted in 1% BSA in PBS. Cells were visualized using a fluorescence microscope (BZ-9000, Keyence).

### Flow cytometry-based LDL uptake assay

iPSC-derived HLCs were treated for 24 h with 5 µM rosuvastatin before the flow cytometry-based LDL uptake assay was performed. iPSC-derived HLCs were dissociated using TrypLE Select (Invitrogen), washed with 1% BSA in PBS, and labelled with primary antibody (ASGPR1; 1:50; Santa Cruz Biotechnology) for 1 h at room temperature. Next, the cells were washed twice with PBS for 5 min each and then incubated for 1 h at room temperature with secondary antibodies (Alexa Fluor 647; 1:500; Invitrogen) diluted in 1% BSA in PBS. Experiments were performed using a Guava EasyCyte Mini Flow Cytometer (Millipore, Billerica, MA, USA). Data analysis was performed using FlowJo software (Tree Star, Inc., Ashland, OR).

### Western blotting

Proteins were extracted using Pierce radioimmunoprecipitation assay buffer (Thermo Fisher Scientific) containing Halt Protease Inhibitor Cocktail (Thermo Fisher Scientific), loaded on a 4%–20% Mini-PROTEAN® TGX™ Gel (Bio-Rad; CA, USA), and then electrophoretically separated and transferred onto polyvinylidene fluoride membranes (Atto-Tec, Tokyo, Japan) using the Trans-Blot Turbo Transfer System (Bio-Rad). The membranes were probed with primary antibodies against LDLR (1:2500; Abcam) and GAPDH (1:2000; Sigma-Aldrich) using an iBind™ Western System (Thermo Fisher Scientific).

### Gene correction of iPSCs using CRISPR/Cas9

Gene correction was performed according to the method of Li *et al*.^29^ with some modifications. To correct the LDLR mutation in HoFH-iPSCs, we constructed a donor template vector using pMA-RQ (ampR) (Thermo Fisher Scientific) in order to replace the mutated sequence with a wild-type sequence harbouring some silent mutations encoding a normal amino acid sequence and a neomycin-selection cassette flanked by two loxP sites (Fig. 1A). We designed a single guide RNA (sgRNA) with a 23-base pair target sequence corresponding to the bases adjacent to the mutation site in exon 6 of human LDLR (underline in Fig. 1B shows the sgRNA binding site) to produce a Cas9-induced double-strand break near the mutation on the LDLR allele using the CRISPR design tool (Thermo Fisher Scientific). We obtained the sgRNA from the template DNA using the TranscriptAid T7 High Yield transcription kit (Thermo Fisher Scientific). A repair oligodeoxynucleotide, containing a T > G correction for the point mutation, was used as a template for the homology-directed repair process induced by Cas9 cleavage (Fig. 1B).

iPSCs were pre-treated with 10 µM Rho-associated protein kinase inhibitor (Y-27632; Sigma-Aldrich) for 1–2 h before transfection. The cells were washed once with PBS, treated with TrypLE Select (Thermo Fisher Scientific) for 3 min at 37 °C, and then washed once with culture medium. Next, they were electroporated with 5 µg of Cas9 nuclease 3NLS (Integrated DNA Technologies, IA, USA), 5 µg of sgRNA, and 10 µg of donor plasmid per 1.0 × 10^6^ cells using a NEPA 21 electroporator (poring pulse: pulse voltage, 125 V; pulse width, 5 ms; pulse number, 2; Nepagene, Ichikawa, Japan).

Cells were plated on a 100-mm dish with neomycin-resistant MEFs (Cell Biolabs, CA, USA) as feeder cells in the presence of 10 µM Y-27632. G418 (50 µg/ml; Nacalai Tesque, Kyoto, Japan) selection was applied after iPSC colonies appeared 48 h post-transfection. The resulting neomycin-resistant colonies were dissociated into single cells and plated at 200–500 cells per 100-mm dish with MEFs. Each subclone was screened by genomic PCR, followed by DNA sequencing. After establishing the single-copy knock-in clones, we electroporated the cells with 10 µg of Cre expression vector pCAG-Cre:GFP, a gift from Connie Cepko (Addgene plasmid # 13776, MA, USA), using a NEPA 21 electroporator as described above.

### Immunogenicity determination by cell-mediated cytotoxicity assay

To confirm the immunogenicity of gcHoFH^+/+^-HLCs against donor PBMCs, we performed a cell-mediated cytotoxicity assay^36,37^. After sampling blood from the donor patient, we separated the extracted PBMCs using Ficoll-Paque PLUS (GE Healthcare Dharmacon Inc., Lafayette, CO). PBMCs were cultured in KBM 502 (Kohjin Bio Co., Sakado, Japan) supplemented with 0.2 µl/ml of Dynabeads™ Human T-Activator CD3/CD28 (Invitrogen) for 5 days.

All iPSC-derived HLCs were loaded with 10 ng/ml of interferon gamma (IFNγ) and incubated overnight at 37 °C in HBM supplemented with 30 ng/ml of oncostatin M and 50 ng/ml of hepatocyte growth factor. For labelling, PBMCs were incubated in PBS with 2 µM CellTrace Violet (Invitrogen) for 4 min at 37 °C. After co-culturing of iPSC-derived HLCs with labelled PBMCs for 24 h in HBM supplemented with 10 ng/ml of IFNγ and 0.5 ng/ml of interleukin 2 (BioLegend), the mixture of floating and attached cells was collected using TrypLE select. The cells were treated with propidium iodide (PI) (Invitrogen). Cell number was counted by flow cytometry using a BD FACS Canto II system (BD Biosciences, NJ, USA). We evaluated HLCs except for PBMCs using CellTrace Violet, and identified PI-positive HLCs as dead cells. Percent cell death represents the number of dead cells divided by the total number of HLCs evaluated. We calculated the mean value of percent cell death in HLC lines that were not co-cultured, and then calculated delta cell death as the difference between percent cell death in each HLC line and this mean value. Data analysis was performed using FlowJo software.

### Statistical analysis

mRNA levels were determined and cell-mediated cytotoxicity assay was performed with three independent biological samples, and the results were expressed as mean ± standard error (SE). Protein expression levels were determined with five independent biological samples, and the results were expressed as mean ± standard error (SE). Statistical analysis was performed using analysis of variance followed by Dunnett’s test for independent samples. Cell-mediated cytotoxicity assay was performed using Tukey-Kramer’s test. The mean difference was considered significant at the p < 0.05 level.

## Supplementary information

Supplemental data
