# Supplementary material for: Function and Immunogenicity of Gene-corrected iPSC-derived Hepatocyte-Like Cells in Restoring Low Density Lipoprotein Uptake in Homozygous Familial Hypercholesterolemia
Source: Sci Rep. 2019 Mar 18;9:4695. doi: 10.1038/s41598-019-41056-w (PMC6423040; doi:10.1038/s41598-019-41056-w)
Supplement: Supplementary file 1 — Supplemental data [file 41598_2019_41056_MOESM1_ESM.docx]

**Function and Immunogenicity of Gene-corrected iPSC-derived Hepatocyte-Like Cells in Restoring Low Density Lipoprotein Uptake in Homozygous Familial Hypercholesterolemia**

Hirofumi Okada, Chiaki Nakanishi, Shohei Yoshida, Masaya Shimojima,

Junichiro Yokawa, Masayuki Mori, Hayato Tada, Tsuyoshi Yoshimuta, Kenshi Hayashi,

Tomoyoshi Yamano, Rikinari Hanayama, Masakazu Yamagishi & Masa-aki Kawashiri

SUPPLEMENTAL DATA

**SUPPLEMENTAL FIGURE & TABLE LEGENDS**

**Supplementary Figure 1. Characteristics of iPSCs and gene-corrected iPSCs from an HoFH patient.** **A**, Pluripotency marker expression was assessed by immunohistochemistry (scale = 100 µm). **B**, PCR to detect expression of pluripotency markers. **C**, Differentiation into three germ layers was monitored by immunofluorescence (scale = 100 µm).

**Supplementary Figure 2. Confirmation that HoFH-iPSCs are derived from donor PBMCs by short tandem repeat analysis.** Electropherogram of two PBMCs lines, WT-PBMCs and HoFH-PBMCs, obtained from two individuals in the D12S391 locus. The STR profile differs between the two cell lines. The STR in the D12S391 locus of HoFH-iPSCs was matched with that in the HoFH PBMCs.

**Supplementary Figure 3. Confirmation of knock-in clones by PCR analysis. A**, Design of primer sets. PCR primers are indicated by arrows. **B**, By PCR analysis using primer set 1, all clones had bands at 240 bp, which indicates that PCR amplification was performed properly. PCR revealed that 13 clones had the knock-in allele (arrowheads). SV40, simian virus 40; Neo, neomycin resistance gene.

**Supplementary Figure 4. Characteristics of iPSC-derived HLCs.** **A**, Bright-field images revealing differentiation of iPSCs into hepatocytes (scale = 100 µm). **B**, Immunostaining analysis for expression of albumin (ALB) and α-1-antitrypsin (A1AT) in WT-iPSC-derived hepatocyte-like cells (WT-HLCs), HoFH-iPSC-derived hepatocyte-like cells (HoFH-HLCs), gcHoFH^+/+^-iPSC-derived hepatocyte-like cells (gcHoFH^+/+^-HLCs), and gcHoFH^+/-^-iPSC-derived hepatocyte-like cells (gcHoFH^+/+^-HLCs) (scale = 100 µm). **C**, RT-PCR analysis of hepatic markers.

**Supplementary Figure 5. Isotype labelling for LDLR.** Immunostaining analysis for expression of LDLR in WT-iPSC-derived hepatocyte-like cells (WT-HLCs) using Isotype control (scale = 50 µm).

**Supplementary Figure 6. Colocalization of LDLR and ER.** Confocal images of HLCs showing dual immunostaining for LDLR (red) together with ER (green) (scale = 50 µm).

**Supplementary Figure 7. Isotype labelling for ASGPR1.** Immunostaining analysis for expression of ASGPR1 in WT-iPSC-derived hepatocyte-like cells (WT-HLCs) using Isotype control (scale = 50 µm).

**TABLE S1. Primer sets for PCR analysis.** Listing of primers used for PCR. Abbreviations: RT-PCR, reverse transcription PCR; gDNA PCR, genomic DNA PCR; F, Forward primer; R, Reverse primer.

**Supplementary Figure 1.**

**Supplementary Figure 2.**

**Supplementary Figure 3.**

**Supplementary Figure 4.**

**Supplementary Figure 5.**

**Supplementary Figure 6.**

**Supplementary Figure 7.**

| Supplemental Table S1. Primer sets for PCR analysis | |
| --- | --- |
| Gene name | Primer sequence (5' - 3') |
| RT-PCR |  |
| NANOG | F: CAGCCCCGATTCTTCCACCAGTCCC |
|  | R: CGGAAGATTCCCAGTCGGGTTCACC |
| OCT3/4 | F: GACAGGGGGAGGGGAGGAGCTAGG |
|  | R: CTTCCCTCCAACCAGTTGCCCCAAAC |
| KLF4 | F: ACGATCGTGGCCCCGGAAAAGGACC |
|  | R: TGATTGTAGTGCTTTCTGGCTGGGCTCC |
| SOX2 | F: GGGAAATGGGAGGGGTGCAAAAGAGG |
|  | R: TTGCGTGAGTGTGGATGGGATTGGTG |
| MYC | F: GCGTCCTGGGAAGGGAGATCCGGAGC |
|  | R: TTGAGGGGCATCGTCGCGGGAGGCTG |
| GAPDH | F: GAGTCAACGGATTTGGTCGT |
|  | R: GACAAGCTTCCCGTTCTCAG |
| SOX17 | F: CGGTATATTACTGCAACTAT |
|  | R: GGATTTCCTTAGCTCCTCCA |
| Hepatocyte nuclear factor 4α | F: CACTCAACGAGAACCAGCAG |
|  | R: TGTCCCGACAGATCACCTC |
| α-1-Fetoprotein | F: AAATGCGTTTCTCGTTGC |
|  | R: GCCACACGGCCAATAGTTTGT |
| Albumin | F: CGCTATTAGTTCGTTACACCA |
|  | R: TTTACAACATTTGCTGCCCA |
| Real-time PCR |  |
| LDLR | F: TCAAGCATCGATGTCAACGG |
|  | R: TGGCACTGAAAATGGCTTCG |
| GAPDH | F: GAGTCAACGGATTTGGTCGT |
|  | R: GACAAGCTTCCCGTTCTCAG |
| gDNA PCR |  |
| Primer set 1 | F: AGGCCCTGCTTCTTTTTCTCTGGT |
|  | R: GCAGCAAGGCACAGAGAATGGGGG |
| Primer set 2 | F: CCTCCTTCCTCTCTCTGGCT |
|  | R: GGGACTTTCCACACCTGGTT |
